# Analysis of prognostic factors in patients with newly diagnosed diffuse large B-cell lymphoma and skeletal involvement

**DOI:** 10.1186/s12885-017-3113-z

**Published:** 2017-02-13

**Authors:** Nicola Lehners, Isabelle Krämer, Maral Saadati, Axel Benner, Anthony D. Ho, Mathias Witzens-Harig

**Affiliations:** 1 Department of Hematology and Oncology, University of Heidelberg, Im Neuenheimer Feld 410, 69120 Heidelberg, Germany; 2 Division of Biostatistics, German Cancer Research Center, Im Neuenheimer Feld 280, 69120 Heidelberg, Germany

**Keywords:** Diffuse large B-cell lymphoma, Bone disease, Prognosis, Risk factors, Radiotherapy

## Abstract

**Background:**

Skeletal involvement (SI) is observed at low prevalence in patients with diffuse large B-cell lymphoma (DLBCL). Due to the rareness of this particular condition, prospective trials for these patients are scarce.

**Methods:**

We analyzed clinical characteristics and outcome of 75 patients with DLBCL and SI in order to identify factors with prognostic impact towards progression-free survival (PFS) and overall survival (OS).

**Results:**

Limited stage disease (Ann Arbor stage IE-IIE) was present in 34 patients (45%), 41 patients (55%) had advanced stage disease (Ann Arbor stage IIIE-IVE). Outcome was generally favorable for patients with DLBCL and SI with 3-year OS of 83%. The international prognostic index (IPI) was able to distinguish between different risk groups within this specific entity. Additionally, hypercalcemia showed to be a factor significantly associated with inferior survival. In regard to first-line treatment modalities, consolidative radiotherapy was positively associated with prolonged PFS and OS while intensification of chemotherapy had no significant impact.

**Conclusions:**

In our cohort of patients with DLBCL and SI, high-risk IPI as well as presence of hypercalcemia were associated with inferior outcome. Consolidative radiotherapy had a positive impact on survival.

## Background

Lymphoma of the bone is a rare entity which encompasses less than 1% of all non-Hodgkin lymphoma [1]. Its most common histologic subtype is diffuse large b-cell lymphoma (DLBCL) [2, 3] with about 50% showing a GCB-phenotype [4, 5]. Clinically, lymphoma of the bone can be classified into three different forms: primary bone lymphoma which consists of a single bone lesion but may involve regional lymph nodes, polyostotic lymphoma which shows multifocal skeletal lesions, and disseminated lymphoma with secondary bone involvement [6].

In regard to prognostic influence, involvement of multiple extranodal sites in lymphoma is generally regarded a risk factor, as reflected in the international prognostic index (IPI) [7, 8]. However, there is few data assessing the prognostic impact of specific extranodal sites or comparing outcome between different extranodal sites [9, 10] and even less on prognostic factors within a particular entity, such as lymphoma with skeletal involvement (SI).

In DLBCL, therapy with R-CHOP (rituximab, cyclophosphamide, doxorubicin, vincristine, prednisolone) is commonly seen as standard of care [11]. Prospective clinical trials optimizing treatment specifically for patients with DLBCL and SI are lacking. Several retrospective analyses see an advantage for combining chemotherapy and radiation treatment [12–14]. Recently, a large meta-analysis of patients with DLBCL and SI treated with CHOP/R-CHOP showed a survival benefit for additional radiotherapy [15]. To the authors’ knowledge there is no published data so far regarding whether intensification of chemotherapy might be beneficial.

In this retrospective analysis, we evaluate the clinical course and treatment strategy of patients with newly diagnosed DLBCL and SI presenting to the University of Heidelberg between 2000 and 2011 with a focus set on the identification of factors particularly relevant for the prognosis of this specific population as well as the impact of different first-line treatment modalities.

## Methods

The clinical database of the University of Heidelberg was reviewed for patients with DLBCL treated at our institution between January 2000 and December 2011. Patients showing SI at first diagnosis were identified and included in this analysis. Presence of SI was determined by means of CT scans. Data on age, sex, Ann Arbor stage, Eastern Cooperative Oncology Group (ECOG) performance status, number of extranodal sites, uni- vs. multifocal SI, serum lactate dehydrogenase (LDH), serum calcium and serum alkaline phosphatase (AP) levels before therapy as well as treatment modalities and outcome were obtained by inspection of medical charts. Progression-free survival (PFS) was defined as time from first diagnosis to the first documentation of progressive disease or death from any cause. Overall survival (OS) was calculated as time from first diagnosis to death from any cause. OS and PFS were estimated using the Kaplan Meier method. Patients lost to follow-up were censored at the time of last follow-up. The impact of the variables cited above on PFS and OS was assessed by univariate regression models after single imputation of missing values. A Cox proportional hazards was used to model PFS and OS in the statistical software R, version 3.0.2. Median follow-up time was calculated using reverse Kaplan-Meier. Furthermore, multivariate proportional hazards regression models were calculated to identify prognostic factors. In all tests, *p*-values < 0.05 were considered statistically significant. This retrospective analysis was approved by the local ethics committee of the University of Heidelberg.

## Results

### Clinical presentation

In total, 821 patients with DLBCL were identified of whom 84 (10.2%) presented with SI at first diagnosis. Nine patients were excluded for further analysis due to prior hematologic malignancy or due to unavailability of any data after establishment of diagnosis, resulting in a study population of 75 patients. Median age of patients with DLBCL and SI was 54 years (range 16–83 years). There was a predominance of men with 51 male patients (68%). The majority of patients (55%) were in advanced clinical stage (Ann Arbor stage IIIE-IVE). More than one extranodal site was present in 51 patients (68%), the most frequent being involvement of skin / soft tissue (39 patients, 52%). Detailed clinical characteristics are given in Table 1.

**Table 1:** Clinical characteristics and first-line treatment modalities

	No. of patients (%) *n* = 75 (100)
Median age [range], years	54 [16–83]
Sex	
Men Women	51 (68) 24 (32)
Ann Arbor stage	
IE-IIE IIIE-IVE B symptoms	34 (45) 41 (55) 26 (35)
LDH	
Elevated Normal Missing	41 (56) 32 (44) 2
ECOG	
0–1 > 1 Missing	50 (72) 19 (28) 6
No. of extranodal sites	
1 2 3 > 3	24 (32) 30 (40) 13 (17) 8 (11)
Calcium	
Elevated Normal Missing	6 (9) 62 (91) 7
AP	
Elevated Normal Missing	14 (22) 50 (78) 11
Skeletal involvement	
Unifocal Multifocal	45 (60) 30 (40)
Chemotherapy	
Palliative CHOP more intensive than CHOP	2 (3) 46 (61) 27 (36)
Rituximab	60 (80)
Radiotherapy	37 (49)
CNS treatment	20 (27)
Autologous transplantation	6 (8)

All patients received chemotherapy. The most common regimen (46 patients, 61%) was cyclophosphamide, doxorubicin, vincristine and prednisolone (CHOP). In 27 patients (36%) a more intensive regimen than CHOP, mainly CHOP plus etoposide or high-dose methotrexate, was applied. Rituximab was part of systemic therapy in 60 patients (80%). Additionally to chemotherapy, 37 patients (49%) received radiotherapy. Six patients underwent autologous transplantation as part of their first-line therapy. For details on treatment regimens refer to Table 1.

After first-line therapy, 39 patients (52%) achieved a complete response, further 25 patients (33%) an unconfirmed complete response or a partial response. Stable disease was noted in 1 (1%), primary refractory disease in 8 patients (11%). Two patients had no formal response evaluation. A total of 18 relapses and 15 deaths of any cause were documented. Survival analysis showed a 3-year PFS of 73% and a 3-year OS of 83%. The median follow-up was 38 months.

### Factors predicting outcome

**Fig. 1:**
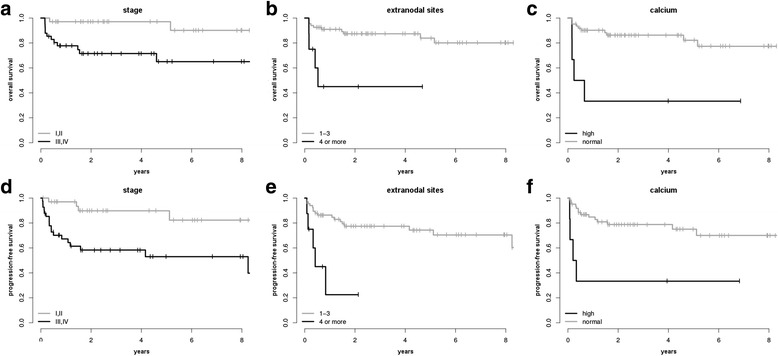
Kaplan-Meier curves for OS (**a**-**c**) and PFS (**d**-**f**) stratified by Ann Arbor stage IIIE/IVE (**a**, **d**), no. of extranodal sites > 3 (**b**, **e**), and hypercalcemia (**c**, **f**). Patients with DLBCL and SI are presented stratified by the three risk factors which showed a significant impact in univariate and multivariate analysis, namely Ann Arbor stage IIIE/IVE, no. of extranodal sites > 3, and hypercalcemia (from *left* to *right*)

To assess the prognostic value of various factors on PFS and OS, we analyzed the individual impact of each of the five IPI components as well as of three bone disease related factors, i.e. uni- vs. multifocal SI, presence of hypercalcemia, serum level of AP. Of the five IPI components, Ann Arbor stage IIIE-IVE, age > 60 years and ECOG > 1 were significantly associated with both inferior OS and PFS in univariate analysis, whereas elevated serum LDH and extranodal sites > 1 did not have any significant influence. However, extranodal sites > 2 showed significant impact towards PFS and extranodal sites > 3 was highly significantly associated with both inferior PFS and OS (*p* = 0.001 and *p* = 0.004, resp.). Of the three additional bone related factors presence of hypercalcemia showed a highly significant impact on PFS and OS (*p* = 0.006 and *p* = 0.0007, resp.) while neither elevated AP nor multifocal bone disease did show any influence. Details of univariate analysis are presented in Table 2 and Fig. 1.

**Table 2:** Univariate analysis of possible prognostic factors and impact of first-line treatment modalities on PFS and OS

	PFS		OS	
	HR [95% CI]	*P*	HR [95% CI]	*P*
Age >60 years	2.85 [1.19, 6.86]	0.02	3.70 [1.22, 11.22]	0.02
Ann Arbor stage IIIE-IVE	4.92 [1.67, 14.52]	0.004	6.41 [1.43, 28.59]	0.01
LDH above normal	2.18 [0.89, 5.33]	0.09	2.29 [0.72, 7.26]	0.16
ECOG >1	3.34 [1.45, 7.70]	0.005	4.27 [1.54, 11.83]	0.005
No. of extranodal sites				
> 1 > 2 > 3	1.24 [0.50, 3.05] 2.64 [1.09, 6.36] 5.69 [1.99, 16.31]	0.64 0.03 0.001	1.11 [0.38, 3.27] 2.56 [0.87, 7.51] 5.91 [1.79, 19.53]	0.85 0.09 0.004
hypercalcemia	4.69 [1.57, 14.03]	0.006	7.52 [2.34, 24.12]	0.0007
AP above normal	0.88 [0.35, 2.16]	0.77	0.71 [0.22, 2.24]	0.56
Multifocal bone lesions	1.10 [0.48, 2.52]	0.83	1.23 [0.44, 3.46]	0.69
Rituximab	0.76 [0.29, 2.00]	0.58	1.12 [0.30, 4.14]	0.87
Intensified chemotherapy	0.44 [0.16, 1.18]	0.10	0.44 [0.12, 1.57]	0.21
Radiotherapy	0.55 [0.20, 1.47]	0.23	0.09 [0.01, 0.72]	0.02

*Abbreviations*: *HR* indicates hazard ratio, *CI* confidence interval, and *P p*-value

In multivariate analysis, number of extranodal sites > 3 was significantly associated with both inferior OS (*p* = 0.04) and PFS (*p* = 0.01); hypercalcemia showed a negative influence on OS (*p* = 0.04), Ann Arbor stage IIIE-IVE on PFS (*p* = 0.01). Details of multivariate analysis are presented in Table 3.

**Table 3:** Multivariate analysis of possible prognostic factors and impact of first-line treatment modalities on PFS and OS

	PFS		OS	
	HR [95% CI]	*P*	HR [95% CI]	*P*
Age >60 years	2.40 [0.90, 6.38]	0.08	1.75 [0.37, 8.24]	0.48
Ann Arbor stage IIIE-IVE	5.65 [1.47, 21.80]	0.01	9.55 [0.87, 104.27]	0.06
LDH above normal	2.16 [0.72, 6.48]	0.17	3.60 [0.56, 23.12]	0.18
ECOG >1	2.80 [0.93, 8.48]	0.07	3.93 [0.78, 20.02]	0.10
No. of extranodal sites >3	5.34 [1.40, 20.32]	0.01	6.81 [1.09, 42.72]	0.04
hypercalcemia	1.76 [0.36, 8.54]	0.48	6.92 [1.09, 44.01]	0.04
AP above normal	0.93 [0.29, 2.97]	0.90	0.67 [0.13, 3.38]	0.63
Multifocal bone lesions	0.35 [0.11, 1.11]	0.08	0.39 [0.08, 1.81]	0.23
Rituximab	0.16 [0.04, 0.62]	0.008	0.23 [0.03, 1.80]	0.16
Intensified chemotherapy	0.50 [0.17, 1.49]	0.21	0.62 [0.13, 2.96]	0.55
Radiotherapy	0.13 [0.04, 0.42]	0.0006	0.01 [0.0006, 0.18]	0.002

*Abbreviations*: *HR* indicates hazard ratio, *CI* confidence interval, and *P p*-value

### Risk scores

**Fig. 2:**
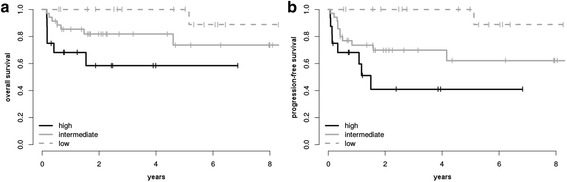
Kaplan-Meier curves for OS (**a**) and PFS (**b**) applying the IPI score. Patients with IPI score <2 are categorized as ‘low’, with IPI score 2 or 3 as ‘intermediate’ and ‘high’ if the IPI is higher than 3

According to IPI risk stratification, 18 patients (24%) were low risk (IPI 0–1), 35 (47%) intermediate risk (IPI 2–3) and 16 (21%) high risk (IPI 4–5). The outcome of patients with low risk IPI was excellent with 3-year PFS of 100% and OS of 100%, whereas patients with intermediate or high risk IPI had a 3-year PFS of 70 and 41% and a 3-year OS of 82 and 58%, respectively (see Fig. 2). Risk stratification applying the IPI score was statistically significant with *p* = 0.002 for PFS and *p* = 0.015 for OS.

We tested whether risk stratification could be improved for the population of patients with DLBCL and SI by utilizing the results of the single risk factor analysis. A new adapted IPI was created including only the IPI factors that had shown significant influence on PFS and OS and adding the highly significant bone related factor hypercalcemia. However, while risk stratification using this new bone score was good, internal validation testing still showed the standard IPI to be superior.

### Influence of first-line treatment modalities

**Fig. 3:**
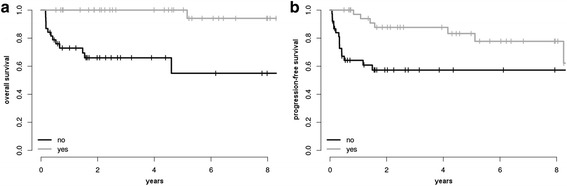
Kaplan-Meier curves for OS (**a**) and PFS (**b**) stratified by radiotherapy

In regard to first-line treatment modalities, the addition of rituximab to chemotherapy did not improve outcome in univariate analysis (*p* = 0.87 for OS, and *p* = 0.58 for PFS). Similarly, escalation of chemotherapy to a more aggressive regimen than CHOP was not significantly associated with prolonged survival (*p* = 0.21 for OS, and *p* = 0.10 for PFS). Consolidative radiotherapy, however, (excluding those patients who were primary refractory to chemotherapy or deceased before first response evaluation and therefore could not undergo adjuvant radiotherapy) had a major impact on OS (*p* = 0.02), yet interestingly did not significantly impact PFS (*p* = 0.23). For details on univariate analysis see Table 2 and Fig. 3.

In multivariate analysis, a positive association was seen between additional rituximab and prolonged PFS (*p* = 0.008), yet not OS. Intensified chemotherapy did not significantly improve outcome. However, consolidative radiotherapy had a major impact on both improved PFS (*p* = 0.0006) and OS (*p* = 0.002).

## Discussion

In this retrospective analysis of patients with newly diagnosed DLBCL with SI, we found an overall favorable outcome with a 3-year OS of 83% and 3-year PFS of 73%. However, patients with Ann Arbor stage IIIE-IVE, hypercalcemia, or no. of extranodal sites > 3 had a significantly inferior survival. Evaluation of the impact of first-line treatment modalities revealed that while consolidative radiotherapy had a positive influence towards prolonged OS, intensification of chemotherapy had no significant impact on survival. However, this analysis was not powered to detect a benefit of intensification of chemotherapy on survival.

Analysis of prognostic factors has led to the development of the IPI [7] for patients with malignant lymphoma with its validity having been confirmed in the rituximab era [8]. For certain sub-entities, however, such as follicular and mantle cell lymphoma, specific scoring systems, i.e. Follicular Lymphoma IPI (FLIPI [16]) and Mantle cell lymphoma IPI (MIPI [17]), have been developed which more accurately distinguish between different prognostic groups in their respective fields. In DLBCL with SI no specific prognostic score exists so far, therefore the IPI is commonly used for these patients. In our cohort of patients with newly diagnosed DLBCL with SI, the IPI was able to separate between groups of low, intermediate and high risk patients. However, looking at the individual risk factors, of the five IPI components, only three factors, i.e. Ann Arbor stage IIIE-IVE, age > 60 years, and ECOG > 1, had significant impact on both PFS and OS in univariate analysis. In multivariate analysis, Ann Arbor stage IIIE-IVE was the only IPI component with significant influence. This is consistent with reports of the favorable outcome of limited stage I-II DLBCL of the bone [14]. In regard to extranodal sites, we found a higher number of extranodal sites, i.e. > 3, to be highly predictive for both inferior PFS and OS. The observation that not all IPI components might be relevant for DLBCL treated with rituximab containing regimens has been made by several authors and different revisions of the IPI were suggested [18, 19]. Similarly, a higher cut-off for the number of extranodal sites was positively evaluated [20]. The latter as well as our findings might imply that not extranodal involvement of every site is necessarily associated with inferior survival. An individual assessment of specific organs involved by DLBCL showed a negative prognostic impact only for involvement of pleura, small intestine, peritoneum, adrenal gland, testis, bone marrow and peripheral blood while SI was not a significant adverse factor in multivariate analysis [9].

As surrogate markers for increased bone metabolism we evaluated the prognostic impact of hypercalcemia, elevated serum AP and multifocal vs unifocal bone lesions. While only few patients showed hypercalcemia, its presence was associated with a very poor outcome. DLBCL presenting with SI and hypercalcemia has been reported as a rare and very aggressive disease [21–23]. Mechanistically, production of osteoclast-activating factors by the lymphoma or its microenvironment seems to be largely responsible for bone destruction and hypercalcemia [24]. Presence of multifocal bone lesions was not associated with inferior survival in our study population. This is consistent with a recent retrospective analysis of multifocal bone DLBCL showing an overall favorable prognosis of this particular entity [25].

In our cohort of patients with DLBCL and SI, the IPI was able to distinguish between different risk groups. However, our risk factor analysis found a different set of variables to have significant impact on survival. These results suggest that for this particular disease entity an improved and more specifically applicable risk score might be achieved. Yet, in our small patient cohort, no newly adapted score could be shown to be superior to the standard IPI in internal validation. Therefore, the development of a specific prognostic score for patients with DLBCL and SI should be attempted in future studies on larger populations.

Analysis of first-line treatment modalities in our patient cohort did not show any difference between patients treated with or without additional rituximab in univariate analysis. However, in multivariate analysis, rituximab seemed to be beneficial in regard to PFS, yet not OS. These slightly conflicting observations might be confounded by the fact that the number of patients in our cohort not receiving rituximab was very small (*n* = 15) compared to those treated with a rituximab containing regimen (*n* = 60) and that they had received treatment predominantly during the earlier years of the time period covered in our study. Therefore, it may not be possible to make reliable conclusions as to the significance of rituximab in the treatment of DLBCL patients with SI in this study. The impact of rituximab on survival of patients with extranodal DLBCL has been controversially discussed with some studies seeing a benefit [3, 26] for the implementation of immuno-chemotherapy while others do not see any such effect [15, 27]. Why rituximab might fail to improve survival of patients with DLBCL and SI has not been satisfactorily explained so far. Thus, there is clearly not sufficient data at this moment to justify withholding this potentially effective treatment option from patients with DLBCL and SI.

Additionally, we analyzed whether escalation of CHOP to a more aggressive regimen was beneficial. Neither in univariate nor in multivariate analysis a significant effect could be seen for this strategy. Consolidative radiotherapy has been positively evaluated in patients with DLBCL [28]. Recently, a large meta-analysis confirmed an event-free survival benefit for patients with DLBCL with SI treated with chemotherapy followed by radiation [15]. In our analysis, consolidative radiotherapy was significantly associated with both prolonged PFS and OS. Therefore, additional radiotherapy after a CHOP-like chemotherapy seems to be recommendable. It is worth mentioning, however, that patients with DLBCL and SI with a low-risk IPI showed an excellent outcome irrespective of the treatment regimen they received.

As a retrospective study this analysis has a number of limitations. The major drawback may be the relatively small number of patients limiting statistical power. This is in part due to the rareness of this particular lymphoma as well as to the fact that this is a single center experience. Furthermore, therapy was not homogenous in the entire study population, as a large time period was covered and treatment options were chosen mostly at the physician’s discretion. To address these difficulties, larger multicenter studies on prognostic factors and therapeutic strategies in this lymphoma entity would be desirable.

## Conclusions

In conclusion, we could show that while patients with DLBCL and SI show a generally favorable outcome, Ann Arbor stage IIIE/IVE, no. of extranodal sites > 3 and hypercalcemia were significantly associated with inferior survival in this cohort. Regarding first-line treatment modalities, escalation of CHOP-like chemotherapy to a more aggressive regimen had no impact on survival while consolidative radiotherapy was associated with prolonged PFS and OS. The latter might be especially relevant for patients in advanced stage or with aggressive disease, e.g. presenting with hypercalcemia. Therefore, exact assessment of the individual prognosis of patients even within rare lymphoma entities is essential in order to allow for individualized therapy.

## Abbreviations

AP: Alkaline phosphatase
CHOP: Cyclophosphamide, doxorubicin, vincristine, and prednisolone
DLBCL: Diffuse large b-cell lymphoma
ECOG: Eastern Cooperative Oncology Group
FLIPI: Follicular lymphoma international prognostic index
IPI: International prognostic index
LDH: Lactate dehydrogenase
MIPI: Mantle cell lymphoma international prognostic index
OS: Overall survival
PFS: Progression-free survival
R-CHOP: Rituximab, cyclophosphamide, doxorubicin, vincristine, and prednisolone
SI: Skeletal involvement

## References

[1] Mikhaeel NG (2012). Primary bone lymphoma. Clin Oncol (R Coll Radiol).

[2] Rathmell AJ, Gospodarowicz MK, Sutcliffe SB, Clark RM (1992). Localised lymphoma of bone: prognostic factors and treatment recommendations. The Princess Margaret Hospital Lymphoma Group. Br J Cancer.

[3] Ramadan KM, Shenkier T, Sehn LH, Gascoyne RD, Connors JM (2007). A clinicopathological retrospective study of 131 patients with primary bone lymphoma: a population-based study of successively treated cohorts from the British Columbia Cancer Agency. Ann Oncol.

[4] Zinzani PL, Carrillo G, Ascani S, Barbieri E, Tani M, Paulli M (2003). Primary bone lymphoma: experience with 52 patients. Haematologica.

[5] Heyning FH, Hogendoorn PC, Kramer MH, Holland CT, Dreef E, Jansen PM (2009). Primary lymphoma of bone: extranodal lymphoma with favourable survival independent of germinal centre, post-germinal centre or indeterminate phenotype. J Clin Pathol.

[6] Messina C, Christie D, Zucca E, Gospodarowicz M, Ferreri AJ (2015). Primary and secondary bone lymphomas. Cancer Treat Rev.

[7] The International Non-Hodgkin's Lymphoma Prognostic Factors Project. A predictive model for aggressive non-Hodgkin’s lymphoma. N Engl J Med. 1993;329(14):987–94. 10.1056/NEJM199309303291402 8141877

[8] Ziepert M, Hasenclever D, Kuhnt E, Glass B, Schmitz N, Pfreundschuh M (2010). Standard International prognostic index remains a valid predictor of outcome for patients with aggressive CD20+ B-cell lymphoma in the rituximab era. J Clin Oncol.

[9] Takahashi H, Tomita N, Yokoyama M, Tsunoda S, Yano T, Murayama K (2012). Prognostic impact of extranodal involvement in diffuse large B-cell lymphoma in the rituximab era. Cancer.

[10] Lopez-Guillermo A, Colomo L, Jimenez M, Bosch F, Villamor N, Arenillas L (2005). Diffuse large B-cell lymphoma: clinical and biological characterization and outcome according to the nodal or extranodal primary origin. J Clin Oncol.

[11] Coiffier B (2005). State-of-the-art therapeutics: diffuse large B-cell lymphoma. J Clin Oncol.

[12] Beal K, Allen L, Yahalom J (2006). Primary bone lymphoma: treatment results and prognostic factors with long-term follow-up of 82 patients. Cancer.

[13] Baiocchi OC, Colleoni GW, Rodrigues CA, Barton D, Kerbauy FR, Garcia RJ (2003). Importance of combined-modality therapy for primary bone lymphoma. Leuk Lymphoma.

[14] Bruno Ventre M, Ferreri AJ, Gospodarowicz M, Govi S, Messina C, Porter D (2014). Clinical features, management, and prognosis of an international series of 161 patients with limited-stage diffuse large B-cell lymphoma of the bone (the IELSG-14 study). Oncologist.

[15] Held G, Zeynalova S, Murawski N, Ziepert M, Kempf B, Viardot A, et al. Impact of rituximab and radiotherapy on outcome of patients with aggressive B-cell lymphoma and skeletal involvement. J Clin Oncol. 2013;31(32):4115-22. 10.1200/JCO.2012.48.0467 24062391

[16] Solal-Celigny P, Roy P, Colombat P, White J, Armitage JO, Arranz-Saez R (2004). Follicular lymphoma international prognostic index. Blood.

[17] Hoster E, Dreyling M, Klapper W, Gisselbrecht C, van Hoof A, Kluin-Nelemans HC (2008). A new prognostic index (MIPI) for patients with advanced-stage mantle cell lymphoma. Blood.

[18] Sehn LH, Berry B, Chhanabhai M, Fitzgerald C, Gill K, Hoskins P (2007). The revised International Prognostic Index (R-IPI) is a better predictor of outcome than the standard IPI for patients with diffuse large B-cell lymphoma treated with R-CHOP. Blood.

[19] Ngo L, Hee SW, Lim LC, Tao M, Quek R, Yap SP (2008). Prognostic factors in patients with diffuse large B cell lymphoma: before and after the introduction of rituximab. Leuk Lymphoma.

[20] Yoo C, Kim S, Sohn BS, Kim JE, Yoon DH, Huh J (2010). Modified number of extranodal involved sites as a prognosticator in R-CHOP-treated patients with disseminated diffuse large B-cell lymphoma. Korean J Intern Med.

[21] Evron E, Goland S, Klepfish A, Malnick SD, Sokolowski N, Sthoeger ZM (1999). Primary multifocal lymphoma of bone presenting as hypercalcemic crisis: report of a rare manifestation of extranodal lymphoma. Leuk Lymphoma.

[22] Takasaki H, Kanamori H, Takabayashi M, Yamaji S, Koharazawa H, Taguchi J (2006). Non-Hodgkin’s lymphoma presenting as multiple bone lesions and hypercalcemia. Am J Hematol.

[23] Matsuhashi Y, Tasaka T, Uehara E, Fujimoto M, Fujita M, Tamura T (2004). Diffuse large B-cell lymphoma presenting with hypercalcemia and multiple osteolysis. Leuk Lymphoma.

[24] Roodman GD (1997). Mechanisms of bone lesions in multiple myeloma and lymphoma. Cancer.

[25] Messina C, Ferreri AJ, Govi S, Bruno-Ventre M, Gracia Medina EA, Porter D (2014). Clinical features, management and prognosis of multifocal primary bone lymphoma: a retrospective study of the international extranodal lymphoma study group (the IELSG 14 study). Br J Haematol.

[26] Pellegrini C, Gandolfi L, Quirini F, Ruggieri P, Stefoni V, Derenzini E (2011). Primary bone lymphoma: evaluation of chemoimmunotherapy as front-line treatment in 21 patients. Clin Lymphoma Myeloma Leuk.

[27] Gutierrez-Garcia G, Colomo L, Villamor N, Arenillas L, Martinez A, Cardesa T (2010). Clinico-biological characterization and outcome of primary nodal and extranodal diffuse large B-cell lymphoma in the rituximab era. Leuk Lymphoma.

[28] Phan J, Mazloom A, Medeiros LJ, Zreik TG, Wogan C, Shihadeh F (2010). Benefit of consolidative radiation therapy in patients with diffuse large B-cell lymphoma treated with R-CHOP chemotherapy. J Clin Oncol.

